# Won’t You be My Neighbor: How Epithelial Cells Connect Together to Build Global Tissue Polarity

**DOI:** 10.3389/fcell.2022.887107

**Published:** 2022-06-21

**Authors:** Lauren E. Cote, Jessica L. Feldman

**Affiliations:** Department of Biology, Stanford University, Stanford, CA, United States

**Keywords:** epithelial fusion, tissue polarity, apical polarity, basal docking, *in vivo*, cell-cell adhesion, lumenogenesis

## Abstract

Epithelial tissues form continuous barriers to protect against external environments. Within these tissues, epithelial cells build environment-facing apical membranes, junction complexes that anchor neighbors together, and basolateral surfaces that face other cells. Critically, to form a continuous apical barrier, neighboring epithelial cells must align their apico-basolateral axes to create global polarity along the entire tissue. Here, we will review mechanisms of global tissue-level polarity establishment, with a focus on how neighboring epithelial cells of different origins align their apical surfaces. Epithelial cells with different developmental origins and/or that polarize at different times and places must align their respective apico-basolateral axes. Connecting different epithelial tissues into continuous sheets or tubes, termed epithelial fusion, has been most extensively studied in cases where neighboring cells initially dock at an apical-to-apical interface. However, epithelial cells can also meet basal-to-basal, posing several challenges for apical continuity. Pre-existing basement membrane between the tissues must be remodeled and/or removed, the cells involved in docking are specialized, and new cell-cell adhesions are formed. Each of these challenges can involve changes to apico-basolateral polarity of epithelial cells. This minireview highlights several *in vivo* examples of basal docking and how apico-basolateral polarity changes during epithelial fusion. Understanding the specific molecular mechanisms of basal docking is an area ripe for further exploration that will shed light on complex morphogenetic events that sculpt developing organisms and on the cellular mechanisms that can go awry during diseases involving the formation of cysts, fistulas, atresias, and metastases.

## Introduction

The fundamental function of epithelial cells is to encase internal tissues to protect them from external challenges while allowing for regulated interactions with the environment. A defining feature of metazoan epithelial tissues is the polarization of cells so that the plasma membrane is divided into an apical surface that faces the “external” environment (e.g., the lumen of an epithelial tube or outside of an epithelial sheet) and a basolateral surface that faces internal tissues [reviewed in (Pickett et al., 2019; Buckley and St Johnston, 2022)]. Cell-cell junctions that reside just below the apical surface tightly link epithelial cells together and provide a selectively permeable barrier [reviewed in (Vasquez et al., 2021)]. Thus, to achieve epithelial integrity, epithelial cells must coordinate apico-basolateral polarity with their cellular neighbors during development, often requiring the coordination of cells from different developmental origins to form functional, continuous epithelial tissues that sustain multicellular life.

**FIGURE 1:**
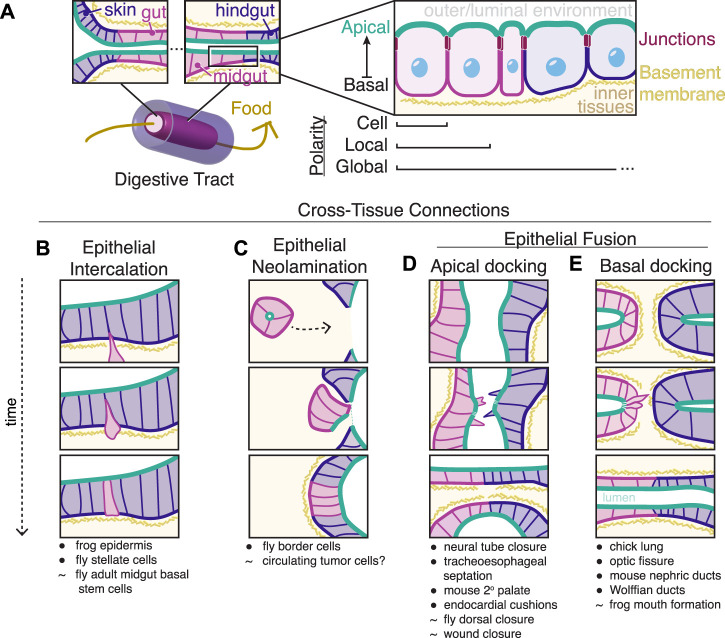
Epithelial tissues connect during development. **(A)** A prominent example of epithelial fusion along an organ system is the digestive tract of the most common body plan of metazoans, a through gut. Outer skin epithelial cells (blue) meet internal gut epithelial cells (pink) at two orifices. These epithelial cells are polarized along an apical (green) to basal axis separated by subapical junctions and lateral cell membranes. This polarity is aligned across multiple scales from individual cells to local groups of cells to global organ systems, including across neighboring tissues. **(B–E)** Simplified diagrams of mechanisms for creating cross-tissue connections. In each case, epithelial neighbors of different origins (blue and pink) connect to create continuous apical surfaces (green). • indicates that the listed process has overall similarity to the diagramed mechanism while ∼ indicates related processes that we or others speculate may use similar mechanisms but where the tissue geometry is substantially different than what is shown in the diagrammed models.

As an example, consider the formation of the most common basic body plan present in bilaterian animals (Dunn et al., 2014), a “through gut” with two orifices opening to the external environment at either end (Figure 1A). At the ends of a through gut, the internal endoderm-derived digestive tissues must connect with external ectoderm-derived epithelial tissues and throughout the digestive tract different tissues (foregut, midgut, hindgut) must connect to create a continuous apical surface. At each of these tissue-tissue interfaces, neighboring cells must align along a common apico-basolateral axis. The ability to form and align such cross-tissue connections correctly is a common requirement in development and defects in these processes can lead to a wide variety of developmental diseases such as oesophageal atresia (van Lennep et al., 2019), spina bifida (Copp et al., 2015), ocular coloboma (Gregory-Evans et al., 2004), congenital abnormalities of the kidney and urinary tract (Murugapoopathy and Gupta, 2020), and persistent cloaca (Escobar et al., 2007). Crohn’s disease patients often develop improper cross-tissue connections (fistulas) between rectal and nearby epithelia (Scharl and Rogler, 2014). The integration of epithelial metastases into foreign tissue (Tsai et al., 2012; Somarelli et al., 2016; Lambert et al., 2017) could also be a type of improper cross-tissue connection.

Connecting different epithelial tissues together requires the coordination of apico-basolateral polarity across multiple scales (Figure 1A). At the scale of the individual cell in an epithelial tissue, an epithelial cell is polarized along its own apico-basolateral axis. A nonadherent apical membrane interacts with the outside or luminal environment and is marked by proteins of the conserved PAR and Crumbs complexes, while the basal surface faces internal tissues and interacts with a specialized extracellular matrix called the basement membrane, primarily made of laminin and type IV collagen. Cell-cell junctions link cells just below the apical surface and include adherens junctions, which contain many transmembrane proteins, most notably E-cadherin, that link actin cytoskeletons across neighboring cells, and occluding (septate or tight) junctions, which form selectively permeable extracellular barriers between adjacent cells (Garcia et al., 2018). While the components of apical, junctional, lateral, and basal complexes are broadly conserved, the initial establishment of apico-basolateral polarity is context dependent (Pickett et al., 2019): for instance, the polarization of the *C. elegans* intestine depends upon the presence of the conserved protein scaffold PAR-3, while the *C. elegans* epidermis can polarize in the absence of PAR-3 (Achilleos et al., 2010). During fly gut development, ectodermally-derived epithelial tissues such as the hindgut, require the apical polarity determinant Crumbs while endodermally-derived midgut cells lose Crumbs expression (Tepass et al., 1990; Campbell et al., 2011) and instead rely on interactions with underlying laminin to correctly polarize (Tepass and Hartenstein, 1994; Yarnitzky and Volk, 1995; Pitsidianaki et al., 2021). Regardless of the mechanism for apico-basolateral polarity establishment within cells, molecular circuits maintain polarity through positive feedback and mutual antagonism of apical, junctional, lateral, and basal protein complexes (Pickett et al., 2019; Buckley and St Johnston, 2022).

Apico-basolateral polarization of an epithelial tissue is only functional if neighboring epithelial cells all orient uniformly to form a continuous barrier. At a local scale within a tissue, epithelial cells coordinate with immediate neighbors to align apico-basolateral axes. At a global scale across a tissue and even across entire organ systems such as a through gut digestive tract, this local polarity must be coordinated into a global apico-basolateral polarity that is consistent across all connected epithelial cells, regardless of their origin or their polarity establishment mechanisms (Figure 1A). Recent *in vivo* work has found that local and global polarity establishment are temporally and genetically separable during the development of the *C. elegans* intestine (Pickett et al., 2021). In the absence of PAR-3, local pockets of apical proteins align between neighboring cells in a HMR-1/E-cadherin-dependent manner, leading to a cystic, non-functional intestine that lacks global polarity (Pickett et al., 2021). Global coordination of apical membrane initiation sites to one common lumen involves the location of the midbody after oriented cell divisions and cadherin-mediated mechanisms that are independent of cell division (Jewett and Prekeris, 2018; Buckley and St Johnston, 2022), as shown in cultured cells (Schluter et al., 2009; Li et al., 2014; Lujan et al., 2016; Liang et al., 2022) and zebrafish development (Tawk et al., 2007; Zigman et al., 2011; Rathbun et al., 2020). The existence of mechanisms to align global polarity that involve cell-cell contact between neighboring cells within one tissue raises the question of how neighboring cells of different tissue types or of different origins ensure alignment of apico-basolateral axes across multiple tissues at a global scale.

### New Neighbors Need Polarity Orientation Information

Complex organ formation requires the connection of cells of different types and origins. While the processes of cell division, cell death, cell extrusion, and lateral intercalation *via* neighbor exchange can bring formerly separated epithelial cells next to one another, these processes typically occur within a field of similar cell types with a common developmental origin. Cells of different types and/or origins become new neighbors by various different ways, some of which are diagrammed in Figures 1B–E and detailed below.

Cells can radially intercalate into an existing epithelium (Figure 1B) to diversify the kinds of cells within the epithelium and to replace old cells (Walck-Shannon and Hardin, 2014). Cells newly joining an epithelium need cues for polarity orientation and/or establishment and these cues can be provided externally by neighboring cells or can arise from within the intercalating cell. In the case of *Drosophila* renal tubes, principal cells (Figure 1B, blue) form an epithelial tube into which secretory stellate cells (Figure 1B, pink) intercalate (Denholm et al., 2003). During this intercalation event, the mesenchymal stellate cells express and localize basal and apical markers only once the cells physically contact the basal and apical domains of the principal cells, respectively (Campbell et al., 2010). In addition to existing epithelia polarizing incoming intercalating cells, existing external structures like the basement membrane can polarize intercalating cells (Chen et al., 2018). In other examples of intercalation, such as in the multiciliated cells of the developing *Xenopus* epidermis, the nascent apical surface is constrained by the geometry of overlying cells and forms an apical surface only at vertices (Stubbs et al., 2006) through directional trafficking of Rab11^+^ vesicles (Kim et al., 2012) and through PAR polarity proteins that promote the formation of apical stabilized microtubules (Werner et al., 2014).

Instead of joining tissue types through the intercalation of individual cells, a migrating cluster of cells can join an epithelium *via* a process recently termed neolamination (Miao et al., 2020) (Figure 1C). Neolamination involves the creation of new, specific, and stable cell-cell contacts, in contrast to delamination or the process of cells losing attachment and leaving an epithelium (Miao et al., 2020). Both cell intercalation and neolamination are closely related to mesenchymal-epithelial transitions (MET) (Pei et al., 2019), during which cells lose mobility and gain clear apico-basolateral polarity. Neolamination refers to one specific step at the very end of a migration event and can describe cells undergoing a MET or cells collectively migrating that never lose apicobasal polarity. In the *Drosophila* egg chamber, border cells (Figure 1C, pink) delaminate from the epithelium at one end of the egg chamber, migrate through the egg chamber as a cluster polarized towards a central apical domain, and finally reorient to an open apical domain that integrates with follicular epithelial cells (Figure 1C, blue) to form a continuous epithelium around the oocyte (Mishra et al., 2019). While the signals that promote the initial rearrangement of polarity in this neolamination process are yet to be determined, the border cells stably attach to the oocyte and follicular cells through noncanonical adhesion by innexins, invertebrate gap junctions (Miao et al., 2020). Speculatively, circulating tumor cell clusters (Aceto et al., 2014; Cheung et al., 2016) may undergo a similar neolamination process upon extravasion out of the blood stream and into new tissues. Supporting this hypothesis, the metastatic potential and survival of circulating cells increases with maintenance of E-cadherin (Padmanaban et al., 2019; Na et al., 2020) and only partial but not full loss of epithelial character (Lüönd et al., 2021), which may correspond to the ability of these cells to create more stable cell-cell attachments than more mesenchymal-like metastatic cells in novel environments.

Epithelial tissues can also join on much larger scales when large sheets of cells fuse, fold, and sort to correctly shape organs. Many classical models of epithelial-to-epithelial connection involve epithelial sheets folding to create internal tubes or are cases of epithelial fusion and sorting (Jacinto et al., 2001; Ray and Niswander, 2012; Fagotto, 2015). In these cases, different epithelial cells initially contact each other at their respective apical or sub-apical/junctional surfaces (Figure 1D): examples include murine secondary palate fusion (Farbman, 1968), endocardial cushion formation (Ghyselinck et al., 1998; Ray and Niswander, 2012), neural tube closure (Pai et al., 2012), and tracheoesophageal separation (Billmyre et al., 2015; Kim et al., 2019; Nasr et al., 2019). Many of these processes may mimic aspects of wound closure or *Drosophila* dorsal closure (Kiehart et al., 2017) in which cells connect while maintaining an outward-facing apical surface that requires minor adjustments within cells to reorient towards a new apical surface. Additionally, actin-rich protrusions are often present at the site of apical fusion (Taya et al., 1999; Jacinto et al., 2000; Rolo et al., 2016). Neighboring tissues fusing at the apical surfaces of contacting cells can bring subapical cell-cell junctions into close proximity to promote correct neighbor-neighbor associations (Nikolopoulou et al., 2017). In the case of neural tube closure, neural-epidermal junctions at the point of fusion are rearranged to neural-neural and epidermal-epidermal junctions to separate the epidermis and neural tube. This junctional exchange at heterotypic neural-epidermal contacts is promoted by localized intracellular Myosin-II contractility in ascidians (Hashimoto and Munro, 2019) or by integrin based adhesions in mice (Escuin et al., 2015; Molè et al., 2020).

**FIGURE 2:**
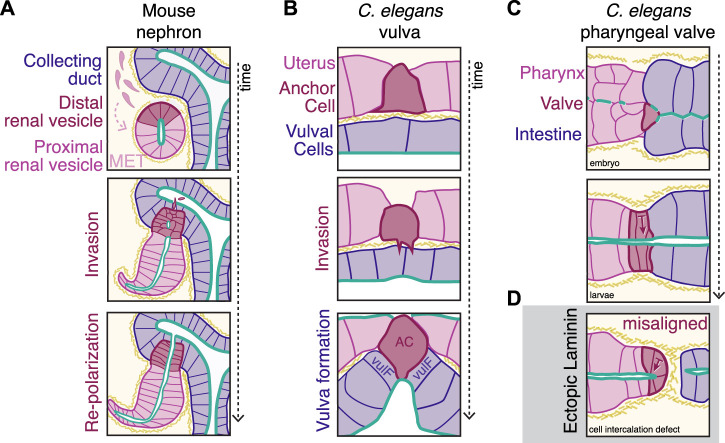
Basal docking connects epithelial tissues that fuse during development. **(A)** Mammalian kidneys develop from the fusion of two epithelial types: the collecting duct (blue, CD) and the renal vesicle (pink/dark red) that forms from a mesenchymal epithelial transition (MET) of the surrounding cells. Cells of the distal renal vesicle (dark red) lose apico-basolateral polarity and invade into the CD, reaching the lumen. These distal cells then repolarize to form a continuous apical connection (green), enabling further elaboration of the nephron structure. Kidney outlines modified from (Kao, 2013). **(B)** The *C. elegans* reproductive tract connects from the uterus (pink) to the vulval epidermal cells (blue, including vulF precursors) via the anchor cell (dark red, AC) that invades through two juxtaposed basement membranes (yellow hashes) over time. **(C)** During development of the *C. elegans* digestive tract, the pharynx (pink) connects to the previously polarized intestine (blue) via the pharyngeal valve cells (dark red) to form a continuous apical (green) midline. **(D)** In animals with cell intercalation defects, ectopic accumulation of laminin (yellow hashes) causes misalignment of valve cell polarity (arrows show basal |—> apical axis).

While apical-to-apical epithelial fusion events are well-studied, recent investigations of development have revealed instances of basal-to-basal epithelial fusion (Figure 1E), including parabronchial fusion in chick lungs (Palmer and Nelson, 2020), vertebrate optic fissure closure (Chan et al., 2020), urogenital development (Pyati et al., 2006; Chia et al., 2011; Slanchev et al., 2011; Weiss et al., 2014; Hoshi et al., 2018; Mello Santos and Hinton, 2019), and vertebrate mouth formation (Soukup et al., 2013; Chen et al., 2017). This arrangement presents unique challenges including the barrier of the basement membrane between tissues and necessary polarity rearrangements upon fusion. Specific dedicated subsets of cells mediate physical tissue fusion from this end-to-end configuration to promote the formation of continuous epithelial surfaces (Figure 2, dark red specialized cells). The remainder of this minireview will focus on examples of these so-called basal docking events and the strategies cells and tissues use to overcome these challenges to maintain global polarity.

### Basement Membrane Is a Barrier to Docking

A key challenge in connecting epithelial tissues that initially meet basal-to-basal and reorient into a continuous epithelium is that joining cells must often contend with at least one layer of basement membrane that separates the tissues. During basal docking, the basement membrane presents a physical barrier between the tissues, and regulated basement membrane breakdown is a key general feature of basal docking between epithelial tissues. In mouse kidney development, loss of the basement membrane between the distal renal vesicle cells and the collecting duct is an early step in connecting these two epithelial tissues (Georgas et al., 2009) (Figure 2A). In the chicken lung, anterior and posterior parabronchial tubules grow towards one another and fuse to form the continuous bronchial tubes required for respiration (Palmer and Nelson, 2020), which involves basement membrane breakdown. Protrusive Wolffian Duct cells contact the cloacal lumen after basement membrane breakdown and then promote localized apoptosis to create the initial plumbing of the murine urogenital tract (Hoshi et al., 2018). During vertebrate eye development, the process of optic fissure closure to create the optic cup involves basement membrane breakdown (Chan et al., 2020). Ocular coloboma, the failure to fuse the retinal epithelium at the optic fissure, is a significant source of human childhood visual impairment, and a zebrafish model of ocular coloboma showed that matrix metalloprotease secretion from the nearby vasculature is required for fusion (Weaver et al., 2020). During *Xenopus* mouth formation, Hedgehog signaling and Wnt antagonists induce basement membrane breakdown between the ectoderm and endoderm (Dickinson and Sive, 2009; Chen et al., 2017). When basement membrane breakdown is blocked during mouth formation, the tissues that would normally connect no longer have a continuous luminal connection, and the resulting tadpoles are unable to feed (Tabler et al., 2014).

The molecular mechanisms that underlie physical basement membrane breakdown, cellular protrusions, and apico-basolateral polarity orientation to promote tissue fusion are best characterized in the *C. elegans* uterine anchor cell, which invades from the uterus into the vulval epithelium during larval development [reviewed in (Hagedorn and Sherwood, 2011; Sherwood and Plastino, 2018)] (Figure 2B). Netrin secretion by the ventral nerve chord (Ziel et al., 2009) polarizes the anchor cell due to clustering of the netrin receptor on the future invasive membrane of the anchor cell at the basement membrane surface (Hagedorn et al., 2009). The polarized anchor cell then invades the underlying vulval epithelium (Sherwood and Sternberg, 2003). The process of invasion involves stabilizing the two juxtaposing uterine and vulval basement membranes (Morrissey et al., 2014), removing basement membrane *via* proteolysis and increasing collagen solubility (Morrissey et al., 2016; Kelley et al., 2019), and forming actin-rich cellular protrusions that are necessary to break through the basement membrane (Caceres et al., 2018). In fact, these cellular protrusions are even strong enough to promote uterus-vulval fusion in the absence of matrix metalloproteases (Kelley et al., 2019).

Although the anchor cell is the major specialized cell that promotes tissue fusion *via* basement membrane breakdown and protrusion formation in this system, the surrounding uterine cells and invaginating vulval cells also play key roles in completing this morphogenetic event. Surrounding cells help enlarge and then stabilize the breach in the basement membrane created by the invasion process. Uterine cells limit trafficking of a basement membrane receptor to the cell surface to promote basement membrane sliding away from the breach, and the underlying invaginating vulval cells stabilize the edge of the breach *via* integrin-based adhesions (Ihara et al., 2011; McClatchey et al., 2016). As the anchor cell invades, it physically deforms the cell membrane of its newly-contacted neighboring vulval cells (vulF cells) to induce lateral membrane constriction (Yang et al., 2017) to promote the reorientation of their apical surface to form a pointed invagination that will become the vulval lumen (Estes and Hanna-Rose, 2009). How adhesion molecules actually mediate the formation of adherens junctions between the anchor cell and the underlying vulval vulF cells after invasion remain unknown, although recent advances in long-term *in vivo* imaging techniques combined with a tissue-specific conditional allele have shown that EGFR signaling within the anchor cell is required to stabilize and align the nascent adherens junctions that connect the anchor cell to the vulF cells (Spiri et al., 2022). Once the anchor cell-vulval connection is complete, the anchor cell fuses with the neighboring uterine cell syncytium (Sapir et al., 2007) to form a thin hymen that is ruptured upon the worm laying its first egg from the internal reproductive tract into the external environment.

### Basement Membrane can Provide Polarity Information

In addition to presenting a physical challenge to basal docking, laminin-rich basement membranes can function in some systems and cell types as a cue to orient, maintain, and even establish apico-basolateral polarity (Lee and Streuli, 2014; Overeem et al., 2015; Matlin et al., 2017). For example, cysts of cultured MDCK cells grown in the absence of basement-membrane components or which lack integrin-mediated signaling have inverted polarity such that apical surfaces face the substrate instead of the lumen, and this defect in polarity orientation can be rescued by the addition of high levels of exogenous laminin (O'Brien et al., 2001; Yu et al., 2005). In the developing murine mammary gland, apical orientation towards a central lumen is actively maintained by β-integrin after it engages with the basement membrane through endocytic removal and polarized trafficking of apical proteins away from the basal surface (Akhtar and Streuli, 2013). In the absence of laminin, but not other basement membrane components, *C. elegans* pharyngeal cells no longer polarize towards a central common midline to form one lumen but rather invert their apico-basolateral polarity, leading to the formation of two lumens (Rasmussen et al., 2012). In developing *Drosophila* midgut, a specific laminin subunit secreted by the underlying mesoderm is required for the re-polarization of migrating endodermal cells (Pitsidianaki et al., 2021). In the adult *Drosophila* midgut, intercalating intestinal stem cells do not require any of the known canonical apical, junctional, or basolateral polarity complexes to establish apico-basolateral polarity, but do rely upon basal integrin signaling to polarize as these cells differentiate into enterocytes and join the intestinal epithelium (Chen et al., 2018).

While these examples show evidence for the role of basement membrane in establishing and maintaining apico-basolateral polarity within individual developing epithelia, other examples show that controlling the initial presence or absence of the basement membrane is crucial for globally aligning fusing epithelial tissues. During *C. elegans* digestive tract development, placement of the basement membrane is important in the connection of the polarized intestine to the polarizing pharyngeal valve cells (Figure 2C). At the connection between the pharynx and intestine, the pharyngeal valve cells normally orient towards a central midline, creating a continuous digestive tract. However, when cell-cell contacts between valve cells and the intestine are abnormal (Figure 2D), ectopic laminin inappropriately surrounds the entire pharynx, and valve cells misorient to close off the lumen and block the remainder of the digestive tract (Rasmussen et al., 2013). Mutations that disrupt normal cell-cell intercalation patterns and gut ablation experiments indicate that E-cadherin-positive contacts between the valve and intestinal cells normally prevent laminin from accumulating at the interface between these two tissues, thereby promoting the correct connection and alignment between the valve and intestine (Rasmussen et al., 2013). Finally, cells of the pharyngeal valve that self-fuse to create a single-celled tube [pm8, vpi1 (Rasmussen et al., 2008)] follow laminin tracts to spread around the midline (Rasmussen et al., 2013), indicating that spatiotemporal patterning of the basement membrane guides the formation of the digestive tract. The use of cell-cell contacts to prevent or promote basement membrane accumulation may be more broadly used to correctly orient cells during cross-tissue connections in multiple contexts and organisms.

### Specialized Cells Guide Basal-to-Basal Docking

The specialized cells that contact tissue ends and connect at basal surfaces during epithelial fusion are different from the rest of the cells within a tissue (dark red cells, Figure 2). A key feature often observed of these specialized cells is the presence of actin-rich protrusions that extend from the basolateral surface and may promote the initial connection between tissues. Some specialized cells maintain aspects of apico-basolateral polarity, such as the preservation of E-cadherin throughout the process of parabronchial tube fusion in the developing chick lung (Palmer and Nelson, 2020). Conversely, in other contexts such as kidney morphogenesis, apico-basolateral polarity is temporarily lost during fusion (Kao et al., 2012; Yang et al., 2013). During basal-to-basal docking of developing chick parabronchial tubes, the cells that mediate epithelial fusion have been identified only by the formation of actin-rich protrusions at the site of basement membrane breakdown (Palmer and Nelson, 2020). How these specific cells send out protrusions while maintaining adherens junctions is unknown, although epithelial cells can send out basolateral protrusions while remodeling junctional contacts (Williams et al., 2014; Walck-Shannon et al., 2015; Sun et al., 2017). Whether the kinds of cellular behaviors present during lateral cell-cell intercalation within tissues (Huebner and Wallingford, 2018) also function during basal docking will be key areas of future exploration.

Kidney morphogenesis is a particularly well-studied example in which a specialized subset of cells facilitates epithelial fusion. Kidney morphogenesis requires the fusion between two tissues, the ureteric collecting duct epithelium and the renal vesicle, a cyst which coalesces as cells undergo an MET (McMahon, 2016). The distal cells of the renal vesicle nearest to the ureteric duct initiate basal docking in a Notch-dependent manner (dark red cells, Figure 2A). Distal cells show a distinct transcriptional profile even from early renal vesicle establishment (Georgas et al., 2009), and lack of distal cells arrests further nephron development after the renal vesicle stage (Kobayashi et al., 2005). Surprisingly, during epithelial fusion with the collecting duct, the distal cells that invade the collecting duct lack apical markers (Kao et al., 2012). After the distal-most cells reach the lumen of the collecting duct, the invading cells repolarize, as seen through the re-expression of E-cadherin, to form local microlumens that eventually combine to form a fully continuous lumen (Georgas et al., 2009; Kao et al., 2012).

Although the identity of these specialized, invasive cells in normal renal development is clear, the cells and/or cues that promote invasive and then repolarization behavior of the distal renal vesicle cells remain unknown (Kao, 2013; Marciano, 2017). Several proteins that influence apico-basolateral polarity in the kidney seem dispensable for parts of the fusion process. Apical continuity and lumen formation in the renal vesicle requires intracellular adherens-junction-associated molecules Afadin (Yang et al., 2013) and p120 catenin (Marciano et al., 2011), however in both cases, removal of either gene in renal vesicle progenitors did not fully inhibit invasion into collecting duct. Cadherin-6 is required globally for robust fusion of the renal vesical to the ureteric bud (Mah et al., 2000), despite being a canonical marker of later proximal cell fate (Cho et al., 1998), and its specific requirement within the distal cells remains untested. Similar to the kidney, investigations of other basal-to-basal docking events have identified putative specific cells that mediate fusion (optic fissure fusion (Bernstein et al., 2018; Eckert et al., 2020), Wolffian duct (Hoshi et al., 2018), nephric duct (Weiss et al., 2014)), however further work is required to understand the specific roles that polarity and adhesion proteins play within these cells during apico-basolateral polarity maintenance, (re)establishment, and alignment.

## Concluding Remarks

To form continuous epithelia of the complicated organs found in metazoans, sheets and tubes must fuse and connect. As mesenchymal or other surrounding tissues are likely crucial to promoting correct epithelial connections (Leung et al., 1999; Weiss et al., 2014; Gestri et al., 2018), these processes must be studied within an *in vivo* context with tissue-specific approaches. Consequently, remarkably little is known about the specific adhesion molecules that mediate different *in vivo* docking interactions. Stable, specific adhesion between two different epithelial tissues is a necessary step in establishing a continuous epithelium with aligned apico-basolateral polarity (Vasquez et al., 2021). One intriguing possibility is that specific adhesive complexes between docking epithelia could provide a mechanism for ensuring that the correct epithelial types find one another. Similar to the neural adhesion code hypothesis that postulates that specific combinations of cell surface receptors specify synaptic connections (Shapiro and Colman, 1999), particular epithelial cells that must dock and undergo tissue fusion could display specific combinations of receptors. The use of different innexins for different steps in neolaminating border cells (Miao et al., 2020) and the Toll receptor code for specifying different boundaries within the *Drosophila* germband epithelium (Paré et al., 2014) are both consistent with the idea that epithelial cell-cell connections are more unique and varied than previously thought.

In returning to the example of the through gut digestive tract, foregut cells must correctly adhere and align with midgut cells on one side and with epidermal cells on the other side. These correct connections across neighbors of different tissues are crucial for organ function and organism survival. Specific epithelial fusion events between different tissues may arise through preferential stabilization of certain cell-cell contacts, just as specific cells sort into separate tissues *via* differential cadherin-based adhesion (Takeichi, 1991; Godt and Tepass, 1998; Price et al., 2002; Tsai et al., 2020), Ephrin-receptor adhesion (Cooke et al., 2005), and capacity to establish stable cell-cell contacts (Skokan et al., 2020). Determining how docking epithelial cells initiate and stabilize adhesions with their neighbors will be critical in the future to determine how epithelial tissues properly align global apico-basolateral polarity and thereby protect organismal integrity.
